# Role of protein kinase C and μ-opioid receptor (MOPr) desensitization in tolerance to morphine in rat locus coeruleus neurons

**DOI:** 10.1111/j.1460-9568.2008.06573.x

**Published:** 2009-01

**Authors:** C P Bailey, J Llorente, B H Gabra, F L Smith, W L Dewey, E Kelly, G Henderson

**Affiliations:** 1 Department of Pharmacy & Pharmacology, University of BathClaverton Down, Bath, UK; 2 Department of Physiology & Pharmacology, University of BristolBristol, UK; 3 Department of Pharmacology & Toxicology, Virginia Commonwealth UniversityRichmond, VA, USA

**Keywords:** desensitization, morphine, opiates, tolerance

## Abstract

In morphine tolerance a key question that remains to be answered is whether μ-opioid receptor (MOPr) desensitization contributes to morphine tolerance, and if so by what cellular mechanisms. Here we demonstrate that MOPr desensitization can be observed in single rat brainstem locus coeruleus (LC) neurons following either prolonged (> 4 h) exposure to morphine *in vitro* or following treatment of animals with morphine *in vivo* for 3 days. Analysis of receptor function by an operational model indicated that with either treatment morphine could induce a profound degree (70–80%) of loss of receptor function. Ongoing PKC activity in the MOPr-expressing neurons themselves, primarily by PKCα, was required to maintain morphine-induced MOPr desensitization, because exposure to PKC inhibitors for only the last 30–50 min of exposure to morphine reduced the MOPr desensitization that was induced both *in vitro* and *in vivo*. The presence of morphine was also required for maintenance of desensitization, as washout of morphine for > 2 h reversed MOPr desensitization. MOPr desensitization was homologous, as there was no change in α_2_-adrenoceptor or ORL1 receptor function. These results demonstrate that prolonged morphine treatment induces extensive homologous desensitization of MOPrs in mature neurons, that this desensitization has a significant PKC-dependent component and that this desensitization underlies the maintenance of morphine tolerance.

## Introduction

The analgesic, respiratory depressant and rewarding effects of morphine occur through activation of μ-opioid receptors (MOPrs; Matthes *et al.*, 1996; Romberg *et al.*, 2003). Tolerance to these *in vivo* responses develops on prolonged exposure (Roerig *et al.*, 1987; Shippenberg *et al.*, 1989; Smith *et al.*, 1999). For many G-protein-coupled receptors prolonged exposure to an agonist results in rapid receptor desensitization through a variety of mechanisms that may involve specific kinases (GRKs) or second messenger kinases, such as protein kinase C (PKC) and A (Kelly *et al.*, 2008). Initial studies suggested that morphine does not induce MOPr desensitization in mature neurons (Alvarez *et al.*, 2002), leading to the suggestion that receptor desensitization was not involved in morphine tolerance (Finn & Whistler, 2001). More recently, however, morphine-induced MOPr desensitization has been observed in mature neurons (Bailey *et al.*, 2003, 2004; Dang & Williams, 2005; Virk & Williams, 2008), although the extent of the desensitization is less than that observed with other opioids.

Our work studying acute MOPr desensitization of recombinant MOPrs and endogenous MOPrs in mature brain neurons has revealed a PKC-dependent component of morphine-induced MOPr desensitization (Bailey *et al.*, 2004, 2006; Johnson *et al.*, 2006), whereas for the opioid peptide, DAMGO, desensitization was almost entirely through a GRK-dependent mechanism (Johnson *et al.*, 2006). There is also evidence to suggest that *in vivo* different opioids induce tolerance by different mechanisms. In GRK3 knockout mice the antinociceptive tolerance that develops to fentanyl was markedly reduced, but that which develops to morphine was not (Terman *et al.*, 2004). A number of studies have reported that PKC is involved in morphine-induced tolerance *in vivo* (Inoue & Ueda, 2000; Zeitz *et al.*, 2001; Bohn *et al.*, 2002; Hua *et al.*, 2002; Smith *et al.*, 2003, 2007). However, such *in vivo* studies do not delineate where in the sequence of intracellular events PKC acts to induce such tolerance.

In this study we have determined the role PKC-induced MOPr desensitization plays in the tolerance to morphine that develops in mature neurons on prolonged exposure to the drug *in vitro* and *in vivo*. To do this we have developed a protocol with which the level of MOPr desensitization can be determined in adult mammalian neurons in the continued presence of the drug. This approach has allowed us to study the time-course of development of MOPr desensitization at the single cell level in brain slices exposed to morphine *in vitro*, and to compare the MOPr desensitization observed *in vitro* with that observed in brain slices prepared from animals chronically treated with morphine (i.e. *ex vivo*). We found that with both *in vitro* and *in vivo* morphine pre-treatment the major component of MOPr desensitization requires ongoing PKC activity to be maintained, whereas incubation of slices with DAMGO induced MOPr desensitization that was not PKC dependent. Analysis of the data using an operational model of drug agonism has allowed us to assess and compare the actual loss of MOPr function that underlies the tolerance that developed to both agonists.

## Materials and methods

### Electrophysiological recordings

#### Brain slice preparation

Male Wistar rats (130–170 g) were killed by cervical dislocation, and horizontal brain slices (200–250 μm thick) containing the locus coeruleus (LC) were prepared as described (Bailey *et al.*, 2003). All experiments were performed in accordance with the UK Animals (Scientific Procedures) Act 1986, the European Communities Council Directive 1986 (86/609/EEC) and the University of Bristol ethical review document.

#### Whole-cell patch-clamp recordings

Slices were submerged in a slice chamber (0.5 mL) mounted on the microscope stage and superfused (2.5–3 mL/min) with artificial cerebrospinal fluid (aCSF) composed of (in mm): NaCl, 126; KCl, 2.5; MgCl_2_, 1.2; CaCl_2_, 2.4; NaH_2_PO_4_, 1.2; d-glucose, 11.1; NaHCO_3_, 21.4; ascorbic acid, 0.1; saturated with 95% O_2_/5% CO_2_ at 33–34°C. For patch-clamp recording LC neurons were visualized by Nomarski optics using infrared light and individual cell somata were cleaned by gentle flow of aCSF from a pipette. Whole-cell voltage-clamp recordings (*V*_h_ = −60 mV) were made using electrodes (3–6 MΩ) filled with (in mm): K-gluconate, 115; HEPES, 10; EGTA, 11; MgCl_2_, 2; NaCl, 10; MgATP, 2; Na_2_GTP, 0.25 (pH 7.3, osmolarity 270 mOsm). Recordings of whole-cell current were filtered at 2 kHz using an Axopatch 200B amplifier and analysed off-line using pClamp.

Activation of MOPrs evoked a transmembrane K^+^ current, and by performing whole-cell patch-clamp recordings a real-time index of MOPr activation could be continually recorded. The opioid-evoked current was continuously recorded at a holding potential of −60 mV. MOPrs and α_2_-adrenoceptors couple to the same set of K^+^ channels in LC neurons (North & Williams, 1985). To reduce variation between cells, the amplitudes of all opioid-evoked currents were normalized to the maximum current evoked by noradrenaline (NA; 100 μm) in the same cell. NA responses were unchanged by any of the drug treatments used in this study. Therefore, any desensitization of MOPrs observed must be homologous to the MOPr. To ensure that responses to NA were mediated through α_2_-adrenoceptors and not attenuated by uptake, NA was always applied in the presence of prazosin (1 μm) and cocaine (3 μm).

All drugs were applied in the superfusing solution at known concentrations. Drugs and chemicals used were from Sigma (Gillingham, UK), except Met-Enkephalin (Bachem, Bubendorf, Switzerland) and Go6976 (Tocris, Bristol, UK). RACK inhibitors were from Kai Pharmaceuticals, San Francisco, CA, USA. The specific RACK inhibitors used were KIG31-1 (a PKCγ inhibitor), KIBI31-1 (a PKCβI inhibitor), KIBII31-1 (a PKCβII inhibitor) and KIC1-1 (a classical PKC isoform inhibitor).

### Induction of morphine tolerance

#### In vitro

Slices were placed on a nylon mesh platform in a pre-incubation chamber containing approximately 250 mL of aCSF at 33–34°C. Given adequate oxygenation, slices incubated in this manner remained viable for up to 12 h. To induce morphine tolerance, morphine (1 or 30 μm) was added to the aCSF bathing the slices for periods of up to 6 h prior to mounting the slices in the recording chamber. The aCSF bathing the slices in the recording chamber also contained morphine at the same concentration as used in the pre-incubation treatment. Whole-cell recordings were then obtained and the slices challenged with opioids up to 9 h after the start of the morphine treatment.

#### In vivo

To induce morphine tolerance rats were injected subcutaneously with 200 mg/kg morphine base contained in a slow-release formulation that contained 200 mg/mL morphine base suspended in an emulsion containing 0.9% NaCl, liquid paraffin oil and mannide monooleate (Arlacel A) in a ratio of 0.5 : 0.42 : 0.08 (v : v : v). Three days after injection of the slow-release morphine the animals were killed and brain slices prepared as described above. This method of morphine treatment has previously been shown to induce significant tolerance to the cellular and analgesic effects of MOPr agonists (Santamarta *et al.*, 2005).

### Data analysis

#### Statistics

For statistical analysis, data are presented as mean ± standard error of the mean, and an unpaired Student’s *t*-test was used to assess significance.

#### Operational model of agonism and loss of receptor function

**Fig. 5:**
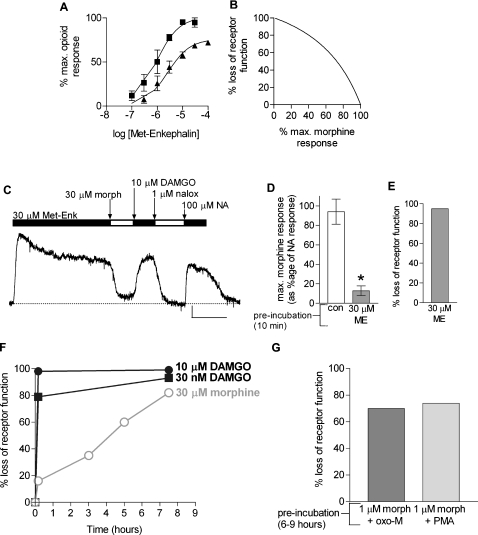
Converting empirical data of morphine responses to loss of receptor function: morphine-induced profound loss of receptor function. (A) Concentration–response curves constructed from the responses to Met-Enkephalin in the absence (squares) and presence (triangles) of the irreversible MOPr antagonist β-FNA, applied at 30 nm for 30 min. Lines of best-fit were obtained following simultaneous linear-regression analysis using the operational model of agonism. (B) Under conditions resulting in loss of receptor function, *τ* would be decreased, resulting in a decrease in the response elicited by 30 μm morphine (% morphine max). Empirical data (response to 30 μm morphine following loss of functional receptor normalized to response to 30 μm morphine under control conditions, i.e. % maximum morphine response) could thus be converted to % loss of receptor function. (C) Met-Enkephalin (Met-Enk; 30 μm) for 10 min resulted in rapid MOPr desensitization. The response to morphine in this desensitized state was 13.7 ± 5.3% that of control (*n*=4; *P < 0.05 vs. control; D), this corresponds to a 95 ± 2% loss of receptor function (E). (F) Empirical data shown in Figs 1 and 4 converted to % loss of receptor function. Black circles, 10 μm DAMGO; black squares, 30 nm DAMGO; open circles, 30 μm morphine. Each point represents mean empirical data converted to % loss of receptor function. (G) Empirical data shown in Fig. 2 converted to percentage loss of receptor function.

The operational model of antagonism (Black *et al.*, 1985) states that
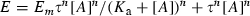
(1) where the response *E* is expressed in terms of the concentration of agonist *A*, the theoretical maximal effect *E*_*m*_ (greater than that which can be functionally attained; Black *et al.*, 1985), the dissociation constant *K*_a_, the transducer ratio *τ* and *n* is the slope of the curve (NB *n* in this equation is not the Hill slope as suggested in Graph Pad Prism). *E*_*m*_ and *n* are intrinsic properties of the receptor/cell and are independent of the agonist used, whereas *τ* depends on the cell, receptor function and agonist used. For the MOPrs in LC neurons, values of *E*_*m*_ and *n* were determined by constructing concentration–response curves to Met-Enkephalin in the absence and presence of the irreversible antagonist β-funaltrexamine (β-FNA; 30 nm for 30 min), and subjecting the curves to simultaneous non-linear regression analysis (see Fig. 5A). Having obtained values for *E*_*m*_ and *n* in this way, then the values of *τ* and *K*_a_ were calculated for each agonist by fitting concentration–response curves by non-linear regression.

**Fig. 1:**
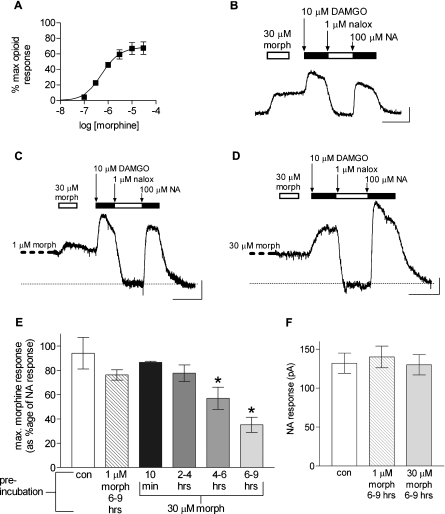
Prolonged exposure to morphine *in vitro* results in MOPr desensitization. (A) Concentration–response curve for morphine in rat LC neurons. Morphine responses were normalized to the maximum possible opioid response in each neuron, evoked by a brief (1 min), application of 10 μm Met-Enkephalin (Met-Enk; *n*=4; error bars represent SEM). (B) Sample current recording from an untreated LC neuron: a receptor-saturating concentration of morphine (30 μm; morph) was applied, followed after 3-min wash by a receptor-saturating concentration of DAMGO (10 μm). The maximum response to morphine was less than that of DAMGO. Following application of the MOPr antagonist naloxone (nalox; 1 μm), a receptor-saturating concentration of noradrenaline (NA; 100 μm) was applied. Scale bars: 50 pA and 5 min. (C) Example recording from an LC neuron, following 6–9 h pre-incubation with 1 μm morphine. Opioid-evoked and NA-evoked currents were elicited using the protocol in (B), by applying 30 μm morphine without washing out the 1 μm morphine in which the slice had been incubated. Naloxone was applied to reveal the baseline current level (dotted line). (D) Example recording following 6–9 h pre-incubation with 30 μm morphine. Note that compared with the NA response, the maximum possible morphine and DAMGO responses were reduced (cf. current traces in B and C). (E) Prolonged treatment with 30 μm, but not 1 μm, morphine reduced MOPr responsiveness as assessed by the maximum response to 30 μm morphine. The effect of 30 μm morphine was time dependent. Pooled data from experiments as described in (C) and (D), displaying responses to 30 μm morphine, normalized to the maximum NA response (*P<0.05 vs. control, Student’s *t*-test; *n*=3–6; error bars represent SEM). (F) Responses to NA following pre-incubation with 1 or 30 μm morphine were unchanged (*n*=3–6; error bars represent SEM).

**Fig. 4:**
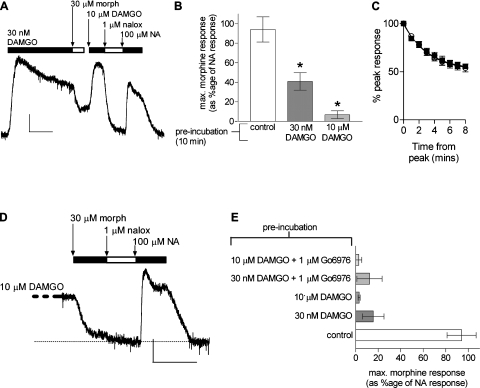
MOPr desensitization induced by DAMGO is PKC independent. (A) Sample current recording showing a 10-min application of DAMGO (30 nm) followed by administration of morphine (morph; 30 μm), 10 μm DAMGO, naloxone (nalox; 1 μm) and noradrenaline (NA; 100 μm) to obtain the maximum opioid and NA responses. Scale bars: 50 pA and 5 min. (B) Pooled data from experiments as shown in (A). DAMGO applied for 10 min at 30 nm or 10 μm caused rapid MOPr desensitization (*P<0.05 vs. control, Student’s *t*-test; *n*=3–6). (C) The acute desensitization of the DAMGO-induced response (shown as a percentage of initial peak response) during an 8-min application of 10 μm DAMGO (open circles) was unaffected by inhibition of PKC with 1 μm Go6976 (black squares). (D) Sample current recording following 6–9 h pre-treatment *in vitro* with 10 μm DAMGO and DAMGO + Go6976 for the last 30–50 min followed by morphine (morph; 30 μm), naloxone (nalox; 1 μm) and noradrenaline (NA; 100 μm) to find the maximum morphine and NA responses. (E) Pooled data from experiments as shown in (D). MOPr desensitization caused by prolonged (6–9 h) DAMGO pre-incubation was not reversed by inhibition of PKC with 1 μm Go6976 (*n*=3–6; error bars represent SEM).

**Fig. 2:**
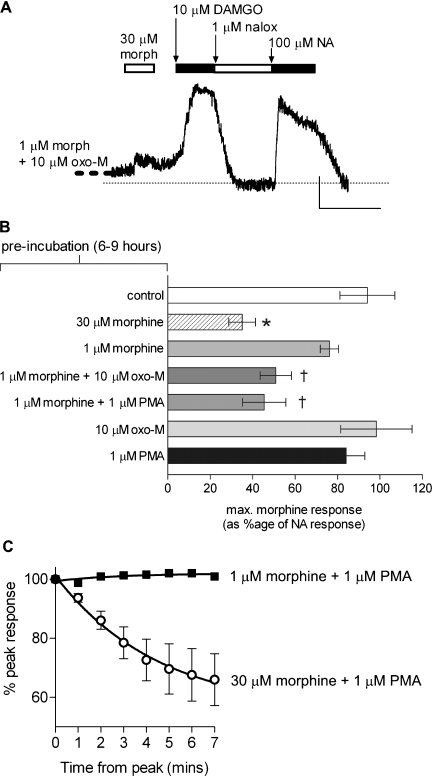
Prolonged exposure to morphine (1 μm) *in vitro* causes MOPr desensitization only when protein kinase C (PKC) is activated**.** (A) Sample current recording from an LC neuron incubated for > 6 h with 1 μm morphine (morph) plus 10 μm oxotremorine-M (oxo-M) followed by the drug protocol described in Fig. 3 (scale bars: 50 pA and 5 min). Naloxone (nalox) was applied to reveal the baseline current level (dotted line). (B) Following 6–9 h pre-incubation with 1 μm morphine alone, there was no decrease in the response to morphine (30 μm) shown as a percentage of the maximum noradrenaline (NA) response. However, when LC neurons were pre-treated with 1 μm morphine and either 10 μm oxo-M or 1 μm phorbol 12-myristate 13-acetate (PMA), the maximum morphine response was significantly decreased (*P<0.05, Student’s *t*-test vs. control; ^†^*P*<0.05, Student’s *t*-test vs. 1 μm morphine alone). Six–nine hours pre-incubation with oxo-M or PMA alone had no effect on opioid responses (*n*=3–6; error bars represent SEM). (C) Morphine (1 μm) in the presence of 1 μm PMA did not induce rapid MOPr desensitization. Black squares: 1 μm morphine + 1 μm PMA. Open circles: 30 μm morphine + 1 μm PMA. Error bars represent SEM.

**Fig. 3:**
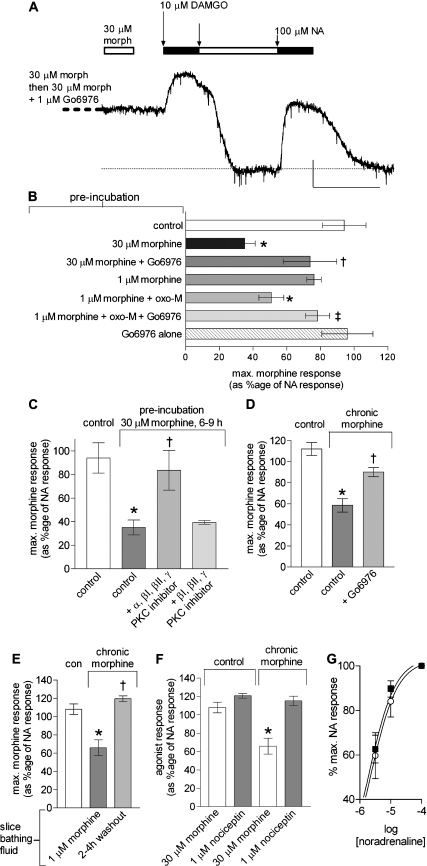
Maintenance of MOPr desensitization during prolonged morphine exposure *in vitro* and *in vivo* requires ongoing PKC activation. (A) Sample current trace from an LC neuron pre-incubated with 30 μm morphine (morph) for > 6 h, followed by 30 μm morphine + 1 μm Go6976 for 30–50 min. Note that the morphine response was elevated compared with Fig. 1D. Scale bars: 50 pA and 5 min. (B) Pooled data from experiments as shown in (A). The decrease in morphine response following pre-incubation with 1 μm morphine and 10 μm oxo-tremorine-M (oxo-M) for 6–9 h was reversed by the addition of the PKC inhibitor, Go6976 (1 μm) for the final 30–50 min of pre-incubation. Similarly, the decreased morphine response following 6–9 h pre-incubation with 30 μm morphine was reversed by Go6976 (1 μm) applied during the final 30–50 min of pre-incubation (*P<0.05 vs. control; ^†^*P*<0.05 vs. 1 μm morphine + oxo-M; ^‡^*P*<0.05 vs. 30 μm morphine, Student’s *t*-test; *n*=3–6). (C) Pre-incubation with 30 μm morphine for 6–9 h resulted in a significant loss of MOPr responsiveness, as seen in Fig. 1E. This effect was completely reversed when all of the conventional PKC isoforms were inhibited for the final 30–50 min of pre-incubation by application of 1 μm of the RACK inhibitor of all conventional PKC isoforms. When 1 μm each of the βI, βII and γ isoforms RACK inhibitors were co-applied, loss of MOPr responsiveness was unaffected (*n*=3–6). All error bars represent SEM (*P<0.05 vs. control pre-incubation; ^†^*P*<0.05 vs. 6–9 h 30 μm morphine pre-incubation). (D) Pooled data from experiments on slices taken from animals pre-treated with morphine for 3 days. Slices from morphine-pre-treated animals were maintained in morphine 1 μm to prevent them going into withdrawal. In these slices the maximum response (i.e. to morphine 30 μm) was significantly decreased compared with that observed in parallel experiments on LC neurons from non-morphine-treated animals. This decrease was significantly reversed by inclusion of Go6976 (1 μm) for the final 30 min before determining the maximum response to morphine. *n*=4–6, error bars represent SEM (*P<0.05 vs. control; ^†^*P*<0.05 vs. chronic morphine). (E) MOPr desensitization in slices taken from morphine-pre-treated animals is reversed if slices are maintained for 2–4 h after slicing in morphine-free bathing solution. *n*= 3–5, error bars represent SEM (*P<0.05 vs. control; ^†^*P*<0.05 vs. chronic morphine, morphine in bathing solution). (F) MOPr desensitization caused by *in vivo* morphine treatment is homologous. In control slices, similar responses are obtained following 30 μm morphine or 1 μm nociception. In slices taken from morphine-pre-treated animals, the nociception response is unchanged, whereas the morphine response is significantly reduced. *n*=3–5, error bars represent SEM (*P<0.05 vs. control animals). (G) Responses to sub-maximal and maximal concentrations of NA in slices taken from morphine-pre-treated animals (open circles) were not different to those observed in slices taken from control animals (black squares; *n*=5; error bars represent SEM).

Following treatments that result in MOPr desensitization, the value of *τ*, which is dependent on the efficacy of agonist–receptor coupling and the number of functional receptors, would be reduced. Therefore, to determine the loss of receptor function resulting from prolonged agonist treatment we measured the response to morphine (30 μm; *E*_2_) after the induction of desensitization. As *E*_*m*_ and *n* are constants, and assuming *K*_a_ to be unchanged, changes in *E* (from *E* to *E*_2_) must therefore depend upon a change in transducer ratio value (from *τ* to *τ*_2_). The percentage loss of receptor responsiveness (*f*) was then calculated (Lohse *et al.*, 1990) using Eq. (2) below.

## Results

### Measuring MOPr desensitization following prolonged drug exposure

To determine the extent of MOPr desensitization resulting from prolonged morphine exposure, we developed a protocol that would enable us to assess the extent of receptor desensitization following prolonged morphine exposure without washing out the morphine as washout of morphine would induce withdrawal and initiate reversal of any desensitization that had been induced. To do this we made use of the fact that morphine is a partial agonist at MOPrs in LC neurons having a lower maximum response than other opioids such as Met-Enkephalin and DAMGO (Fig. 1A and B; see also Alvarez *et al.*, 2002; Bailey *et al.*, 2003). A basic tenet of receptor theory is that for a partial agonist the maximum response is produced only when all of the available receptors are occupied, and therefore any loss of MOPr function as would occur if receptors were desensitized would result in a decrease in the maximum response to the partial agonist. Therefore, any deficit in receptor function caused by drug treatments would be seen as a decrease in the maximum response to morphine (measured by applying a receptor-saturating concentration, 30 μm, of morphine).

In preliminary experiments on drug-naïve slices we demonstrated that a receptor-saturating concentration of morphine (30 μm) evoked a K^+^ current that was 94 ± 13% of the maximum response evoked by NA in the same neurons (Fig. 1B). In all our experiments the opioid responses have been normalized to the maximum NA response (NA; 100 μm) mediated through α_2_-adrenoceptors in the same neuron to control for variations in current amplitudes between neurons. MOPrs and α_2_-adrenoceptors couple to the same set of GIRK channels in LC neurons (North & Williams, 1985). Following washout of the morphine for 3 min, application of a receptor-saturating concentration of the full agonist, DAMGO (10 μm), evoked a response of 149 ± 15% of the maximum response to NA. These values for the maximum responses evoked by morphine and DAMGO administered sequentially to the same neurons are not different from those in slices where neurons were exposed to only one of the opioids. This demonstrates that it is possible in the same neuron to obtain maximum response amplitudes for morphine and DAMGO.

### MOPr desensitization following prolonged exposure to morphine in vitro

Slices containing the LC were incubated for 6–9 h in 1 or 30 μm morphine or control aCSF, and whole-cell patch-clamp recordings were performed under the same conditions. Morphine (1 μm) was chosen as it is a sub-maximal concentration of morphine, and has been shown previously to approximate to the concentration measured in rat brains following a moderate analgesic dose of morphine (Patrick *et al.*, 1978). Morphine (30 μm) was chosen as it is a receptor-saturating concentration of morphine. The degree of MOPr desensitization induced by each concentration of morphine was then assessed.

Figure 1C and D shows sample traces from LC neurons in slices that had been incubated for 6–9 h in 1 μm or 30 μm morphine, and then maintained in the continued presence of morphine whilst the recording was initiated. Morphine (30 μm) and DAMGO (10 μm) were then applied sequentially to obtain the maximum responses to each drug, and naloxone (1 μm) applied to return the holding current to the true baseline level from which opioid-evoked current amplitudes could be measured. Pooled data (Fig. 1E) show that 6–9 h pre-incubation in 1 μm morphine alone caused no statistically significant decrease in the maximum response to morphine, compared with data from slices incubated in control aCSF for 6–9 h. In contrast, when LC slices were incubated with 30 μm morphine for periods up to 6–9 h there was a gradual decrease in the maximum response to morphine over time that first became statistically significant at the 4–6 h time-point (Fig. 1E); after 6–9 h pre-incubation the maximum response to morphine had decreased by 63 ± 7%. Note, unlike rapid MOPr desensitization (Bailey *et al.*, 2004), that produced by prolonged exposure to 30 μm morphine was observed without the need to activate PKC with oxotremorine-M (oxo-M) or phorbol 12-myristate 13-acetate (PMA). To ensure that the desensitization induced by the prolonged exposure to 30 μm morphine had not in some non-specific way interfered with G-protein coupling or potassium channel function, we also measured the amplitude of the maximum NA-evoked current. There was no change in the amplitude of the maximum NA-evoked current following exposure of LC slices to either 1 or 30 μm morphine for up to 9 h (Fig. 1F and Supporting Information Fig. S1, A). It is concluded therefore that exposure of LC neurons *in vitro* to 30 μm but not 1 μm morphine, alone for 6–9 h, induces significant homologous MOPr desensitization.

Because 1 μm morphine alone did not induce MOPr desensitization, we next examined the effect of PKC activation on the development of MOPr desensitization during prolonged exposure to 1 μm morphine. Slices were incubated for 6–9 h with morphine (1 μm) as well as oxo-M (10 μm) to activate PKC. In control experiments 6–9 h incubation with oxo-M alone had no effect on the maximum response to morphine, whereas following exposure to morphine plus oxo-M the maximum response to morphine was decreased to 46 ± 8% (Fig. 2A and B). The effect of oxo-M could be mimicked by activating PKC with the phorbol ester, PMA. When slices were incubated with both morphine (1 μm) and PMA (1 μm), the maximum response to morphine was reduced by 52 ± 10% (Fig. 2B). Like oxo-M, PMA alone (6–9 h) had no effect. Thus, MOPr desensitization can be induced by prolonged exposure to 1 μm morphine *in vitro*, but only when the PKC activity in LC neurons is enhanced above the basal level in isolated neurons. The decrease in responsiveness was selective to the MOPr, as the NA responses were unaffected by any pre-treatment (Supporting Information Fig. S1, C). In acute exposure (7 min) experiments, 1 μm morphine did not induce MOPr desensitization in the absence or presence of PKC activation by PMA (Fig. 2C), indicating that, even when PKC is activated, 1 μm morphine causes MOPr desensitization only after prolonged exposure to the drug.

Whilst prolonged exposure to morphine 30 μm alone, or morphine 1 μm+ oxo-M or PMA, reduced the maximum response to morphine and thus revealed a loss of functional MOPrs, only 30 μm morphine pre-treatment resulted in a decrease in the maximum response to the full agonist DAMGO (Supporting Information Fig. S1, B and D). As a high-efficacy agonist, DAMGO has a significant spare receptor reserve in LC neurons (Osborne & Williams, 1995), such that, in comparison to the partial agonist morphine, a greater amount of MOPr desensitization is required to produce a decrease in the maximum DAMGO response.

### Role of PKC in maintaining MOPr desensitization during prolonged *in vitro* exposure to morphine

To examine whether ongoing PKC activity was required to maintain MOPr desensitization, we investigated whether PKC inhibitors could reverse morphine-induced MOPr desensitization after desensitization had already been induced. Slices were first incubated with 1 μm morphine + 10 μm oxo-M for 6–9 h, and then the conventional PKC isoform inhibitor Go6976 (1 μm) was added for 15–30 min prior to initiation of whole-cell recording, and for a further 15–20 min prior to determining the maximum responses to morphine (i.e. the neurons were only exposed to the PKC inhibitor for the last 30–50 min of the 6–9-h prolonged morphine exposure). Figure 3A and B shows that the presence of Go6976 for the final 30–50 min of incubation with morphine + -M significantly reversed the MOPr desensitization induced by morphine. Go6976 alone had no effect on the maximum response to morphine (Fig. 3B).

Having shown that MOPr desensitization to 1 μm morphine required PKC activation, we next tested the hypothesis that the desensitization induced by 30 μm morphine alone was because there was sufficient basal PKC activity to cause MOPr desensitization by this receptor-saturating concentration of morphine. We incubated slices with 30 μm morphine for 6–9 h, followed by addition of Go6976 (1 μm) in the continued presence of morphine for 30–50 min (i.e. prior to and during the recording period). Go6976 significantly reversed the tolerance induced by 30 μm morphine alone (Fig. 3B).

We next examined which PKC isoform(s) could be involved in the MOPr desensitization induced by prolonged exposure (6–9 h) to 30 μm morphine alone using RACK inhibitors (Chen *et al.*, 2001). We first used a RACK inhibitor that blocks all conventional PKC isoforms (i.e. PKCα, βI, βII and γ), but does not block novel (i.e. PKCδ, ε, θ and μ) or atypical PKC isoforms (i.e. PKCξ and λ). Exposure of slices to a RACK inhibitor that blocks all conventional PKC isoforms (i.e. PKCα, βI, βII and γ; Ron *et al.*, 1995) for 30–50 min at the end of the prolonged exposure to morphine significantly reversed the MOPr desensitization induced by 30 μm morphine (similar to when conventional PKC isoforms were inhibited by Go6976), whereas a combination of RACK inhibitors that block PKCβI, βII and γ (Johnson *et al.*, 1996; Stebbins & Mochly-Rosen, 2001) did not (Fig. 3C). These data indicate that PKCα is probably the isoform required to maintain prolonged morphine-induced MOPr desensitization.

For both 1 μm morphine + oxo-M and 30 μm morphine alone, the MOPr desensitization was reversed when the PKC inhibitors were added after 6–9 h of morphine exposure had already occurred and thus MOPr desensitization had already been initiated. This demonstrates that ongoing PKC activity is essential to maintain MOPr desensitization.

### Role of PKC in maintaining MOPr desensitization following in vivo exposure to morphine

Slices were prepared from rats pre-treated *in vivo* with morphine for 3 days (see Materials and methods). During all stages of slice preparation the fluid bathing the brain contained morphine (1 μm). In LC neurons from morphine-pre-treated rats the maximum response to morphine (30 μm) was significantly lower than that observed in parallel experiments on LC slices from untreated animals and not incubated in morphine (Fig. 3D). In neurons from morphine-pre-treated animals the maximum response to morphine was decreased by 48%. In these slices the maximum response to NA (156 ± 15 pA) was not significantly different from the maximum NA response evoked in slices from non-morphine-pre-treated animals (Supporting Information Fig. S1, A), suggesting that the MOPr tolerance observed was homologous. To test this further, we examined whether chronic morphine treatment caused ORL1 desensitization, a receptor that has been shown to desensitize in a PKC-dependent manner (Pu *et al.*, 1999). We saw no effect on ORL1 function following *in vivo* morphine treatment, further suggesting that the MOPr desensitization was homologous (Fig. 3E).

To examine whether ongoing PKC activity was required to maintain the MOPr desensitization induced by *in vivo* exposure to morphine, slices were prepared as above and incubated in morphine (1 μm). Whole-cell recordings were obtained and the conventional PKC isoform inhibitor Go6976 (1 μm) was added for 30 min prior to determining the maximum response to morphine. The presence of Go6976 significantly, but not completely, reversed the MOPr desensitization induced by the *in vivo* morphine pre-treatment (Fig. 3D). As well as ongoing PKC being required to maintain MOPr desensitization, the MOPr needs to be bound by morphine. When morphine was removed from the slice-bathing solution for 2–4 h, chronic morphine-induced MOPr desensitization was completely reversed (Fig. 3F). The MOPr desensitization induced by chronic *in vivo* morphine treatment was homologous in that responses to sub-maximal and maximal concentrations of NA were unaffected (Fig. 3G).

### PKC is not involved in the MOPr desensitization following prolonged in vitro exposure to DAMGO

To examine the MOPr desensitization induced by DAMGO, we used two concentrations of the drug, 30 nm and 10 μm. DAMGO (30 nm) is a sub-maximal concentration of the drug, being an approximate EC_70_ concentration (i.e. it produces an equivalent amplitude of response to 30 μm morphine), whereas 10 μm DAMGO is a receptor-saturating concentration. Both concentrations of DAMGO induced profound rapid MOPr desensitization (Fig. 4A–C). By applying morphine (30 μm) immediately following exposure to DAMGO we could again measure the extent of MOPr desensitization by the decrease in the maximum response to morphine. Acute (10 min) exposure to DAMGO 30 nm and 10 μm both produced marked decreases in the maximum response to morphine (Fig. 4B). Rapid MOPr desensitization by DAMGO was unaffected by the PKC inhibitor Go6976 (1 μm; Fig. 4C).

MOPr desensitization persisted after prolonged (6–9 h) exposure to DAMGO 30 nm or 10 μm (Fig. 4D and E). Incubation of the slices with Go6976 (1 μm) for the last 30–50 min of DAMGO pre-incubation did not inhibit the MOPr desensitization induced by either concentration of DAMGO (Fig. 4E). Therefore, MOPr desensitization induced by prolonged DAMGO exposure was not mediated by PKC.

### Loss of receptor function underlying MOPr desensitization by different agonists

We have used the operational model of agonism [Eq. (1) above;Black *et al.*, 1985; ] to determine the actual loss of MOPr function that is responsible for the levels of desensitization observed. This is important because the actual loss of receptor function required to reduce the maximum response to a full agonist may be much greater than that required to cause a similar reduction in the maximum response to a partial agonist as a full agonist will have a receptor reserve that must be removed before any loss of maximum response is observed (Connor *et al.*, 2004).

Figure 5A shows concentration–response curves to Met-Enkephalin in control and in neurons pre-treated with the irreversible MOPr antagonist β-FNA, with the lines of best-fit derived from the operational model of agonism. In LC neurons, the values for MOPr activation are: *E*_m_ (theoretical maximum response) = 115%; *n* (slope of the curve; NB not the Hill slope) = 0.96. For morphine (see concentration–response curve in Fig. 1A), the *K*_a_ (dissociation constant) = 1.6 μm and *τ* (the transducer ratio) = 1.60. These values are similar to those reported by Osborne & Williams (1995) for MOPr activation in LC neurons.

This then enables us to derive a model (Fig. 5B) whereby the functional change in the amplitude of the response to morphine (30 μm) resulting from MOPr desensitization (*x*-axis, Fig. 5B) can then be used to calculate new values for *τ* and, using Eq. (2), thus calculate the actual loss of MOPr function (*y*-axis, Fig. 5B; see Materials and methods for details and definitions). The percentage loss of receptor function (*f*) is given by: 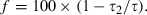
(2)

For example, the MOPr desensitization induced by acute exposure to Met-Enkephalin (30 μm; Fig. 5C and D) reduced the response to 30 μm morphine to 14 ± 5% of control (*n*=4; Fig. 5E). Using the methods described above we calculated that this translated to a 95% loss of MOPr function, a finding that corresponds well to a previous estimate by Osborne & Williams (1995). We have then gone on to convert the empirical data in Figs 1–4 to % loss of MOPr function.

Acute exposure of neurons to DAMGO very rapidly induced a high loss of MOPr function, such that after only a 10-min exposure to 30 nm DAMGO or 1 μm DAMGO there was a 79% and 98% loss of MOPr function, respectively (Fig. 5F). The loss of MOPr function induced by acute morphine was less, even in the presence of PKC activation. Thus, a 7-min exposure to morphine (1 μm) in the absence or presence of PMA induced no loss of MOPr function (see Fig. 2C), whereas over the same time period 30 μm morphine in the presence of PMA reduced MOPr function by 53%.

.

Treatment for 6–9 h with 1 μm morphine alone caused no significant decrease in MOPr function; however, when neurons were incubated with 1 μm morphine and either oxo-M (10 μm) or PMA (1 μm), receptor function was decreased by 70% and 74%, respectively (Fig. 5G). Prolonged *in vitro* exposure to 30 μm morphine alone did cause profound loss of MOPr function (Fig. 5F), but this occurred more slowly than with DAMGO. Following 6–9 h treatment with 30 μm morphine, there was an 82% loss of MOPr function, which compares to that induced by acute (10 min) DAMGO. In neurons from animals pre-treated with morphine, MOPr receptor function was decreased by 71%, which is the same as that observed with *in vitro* morphine plus PMA or oxo-M treatment.

It appears therefore that exposure to DAMGO induces MOPr desensitization that is both rapid in onset and extensive, whereas morphine-induced MOPr desensitization develops more slowly, requires ongoing PKC activation, but both *in vitro* and *in vivo* morphine treatment can induce a similar level of loss of receptor function.

## Discussion

A central finding of this study is that prolonged exposure of mature neurons to morphine causes profound MOPr desensitization. It had been suggested that morphine produces tolerance because it does not induce rapid MOPr desensitization and that morphine tolerance occurs following subsequent, as yet unknown, adaptive changes caused by prolonged MOPr signalling (Finn & Whistler, 2001). Our data would refute that hypothesis. Morphine does induce homologous MOPr desensitization, following prolonged *in vitro* or *in vivo* morphine treatment. This finding re-confirms the view that MOPr desensitization contributes to morphine tolerance.

To circumvent problems in the interpretation of opioid agonist-induced desensitization data (reviewed in Connor *et al.*, 2004), we specifically designed our experiments so that we could investigate agonist coupling and desensitization immediately following long-term *in vitro* or *in vivo* morphine treatments, in the absence of complications due to drug withdrawal. Indeed, after 2–4 h morphine withdrawal *in vitro*, MOPr desensitization was reversed. This suggests that the MOPr desensitization we observed was somewhat transient, in contrast with that seen by Christie *et al.* (1987) where MOPr desensitization was still seen in rat LC neurons up to 6 h after morphine withdrawal *in vitro*. In that study the chronic morphine treatment used higher doses than in this study (> 2700 mg/kg morphine pellets over 7 days vs 200 mg/kg emulsion over 3 days), and possibly in their experiments the level of chronic treatment induced additional adaptive mechanisms, such as enhanced GRK and arrestin activity (Terwilliger *et al.*, 1994) or receptor downregulation that may result in longer-lasting MOPr desensitization.

Further, there have been recent studies in LC and periaqueductal grey neurons demonstrating that, following *in vitro* withdrawal from chronic morphine treatment, MOPrs exhibit enhanced agonist-induced desensitization (Dang & Williams, 2005; Ingram *et al.*, 2008). These studies suggest the possibility that morphine withdrawal and/or higher levels of morphine tolerance may induce additional adaptive mechanisms, such as effects on GRKs or RGS proteins (Terwilliger *et al.*, 1994; Gold *et al.*, 2003).

Our data with PKC inhibitors reversing morphine-induced desensitization appear somewhat at odds with those of Dang & Williams, 2005. In their paper, using sharp electrode recording, they observed that a relatively high concentration of chelerythrine enhanced the desensitization induced by morphine-6-glucuronide. We have shown in this paper and previously (Bailey *et al.*, 2004) that a range of structurally different PKC inhibitors reverse morphine-induced desensitization.

To obtain a quantitative measure of functional MOPr loss, we subjected the data to operational analysis (Black *et al.*, 1985) to obtain a quantitative measure of functional MOPr loss. This revealed that prolonged exposure to morphine both *in vitro* and *in vivo* resulted in up to an 82% loss in MOPr function (Christie *et al.*, 1987). Although the desensitization induced by morphine was slightly less than that induced by DAMGO and developed over a longer time period, it is evident that prolonged morphine exposure can induce robust loss of MOPr function in adult mammalian neurons. Importantly, MOPr desensitization occurs at the level of the MOPr and not downstream of the receptor, as the NA-induced K^+^-current in the same neurons was unaffected. α_2_-Adrenoceptors and MOPrs couple to the same set of K^+^ channels in LC neurons (North & Williams, 1985).

Inhibitors of PKC significantly reversed MOPr desensitization even after the desensitization had been allowed to develop (over 3 days of morphine treatment *in vivo* and 6–9 h morphine treatment *in vitro*). This shows that for morphine desensitization to persist, ongoing PKC activity is required. Crucially, this finding is paralleled in previous studies of morphine analgesia tolerance *in vivo* where PKC inhibitors were able to reverse tolerance even when they were first administered after 3 days of morphine exposure (Smith *et al.*, 1999). Furthermore, *in vivo* morphine analgesia tolerance can be potentiated by a PP2A phosphatase inhibitor, indicating that the level of tolerance is dependent upon the balance between ongoing MOPr phosphorylation and dephosphorylation by PP2A (Gabra *et al.*, 2007).

PKC exists as multiple isoforms (Way *et al.*, 2000), many of which are expressed in CNS neurons. In LC neurons a single conventional PKC isoform, PKCα, appears to be responsible for the PKC component of morphine-induced MOPr desensitization. However, PKCα is not the only isoform that may be involved in morphine-induced MOPr desensitization, and it is entirely possible that in different neuronal populations other isoforms of PKC may be involved. Indeed, *in vivo*, there is evidence from analgesia studies that morphine tolerance may involve not only PKCα but also PKCε and PKCγ (Zeitz *et al.*, 2001; Hua *et al.*, 2002; Newton *et al.*, 2007; Smith *et al.*, 2007).

In LC neurons DAMGO induces rapid (*t*_1/2_=150 s) MOPr desensitization (Harris & Williams, 1991; Bailey *et al.*, 2003). We have reported that in HEK-293 cells rapid DAMGO-induced MOPr desensitization is GRK-mediated because it is blocked by overexpression of a GRK2 dominant negative mutant (Johnson *et al.*, 2006). In the present experiments we sought to determine whether, on prolonged exposure to DAMGO, a PKC component of MOPr desensitization was revealed. This does not appear to be the case because treatment with a PKC inhibitor that blocked morphine-induced MOPr desensitization failed to alter the DAMGO-induced desensitization following 6–9 h pre-treatment with DAMGO. Furthermore, it might have been expected that DAMGO would induce less MOPr desensitization than morphine given that DAMGO induces not only MOPr desensitization but also MOPr internalization that would result in MOPr resensitization by dephosphorylation and then re-insertion of reactivated receptors into the plasma membrane, whereas morphine induces much less MOPr internalization. However, we found that a receptor-saturating concentration of DAMGO induced an almost complete loss of MOPr function.

In LC neurons *in vitro* under conditions of low PKC activity, a component of morphine-induced rapid MOPr desensitization persists (Bailey *et al.*, 2004; Dang & Williams, 2005; Virk & Williams, 2008). This could be because there is, in addition to PKC-mediated desensitization, a component of morphine desensitization that is GRK-mediated. This might also explain why, in our *ex vivo* experiments where neurons had been exposed to morphine for 3 days, the PKC inhibitor did not completely reverse MOPr desensitization, and why a small, but significant amount of receptor internalization was induced by morphine in HEK-293 cells (Johnson *et al.*, 2006) and striatal neurons (Haberstock-Debic *et al.*, 2005).

The present results, together with our previous work (Bailey *et al.*, 2004; Johnson *et al.*, 2006), reveal that there are four main factors that determine the development of MOPr desensitization: the nature of the agonist ligand; the degree of receptor occupancy; the duration of morphine treatment; and the level of PKC activation. LC neurons in the slice preparation can be considered to be in a ‘basal state’, where PKC activity is lower than in the same neurons *in vivo*, as *in vivo* ongoing synaptic activity will activate Gq-coupled metabotropic receptors and Ca^2 +^-permeable ionotropic receptors that would elevate PKC activity. Prolonged exposure to a sub-maximal, therapeutically relevant concentration of morphine (1 μm) induced MOPr desensitization *in vitro* only when PKC activity was elevated. In contrast, when slices were prepared from morphine-pre-treated rats and maintained in this concentration of morphine, profound MOPr desensitization was observed that could be reversed by inhibiting PKC. Comparable levels of MOPr desensitization in the brain have been reported using agonist-induced [^35^S]GTPγS autoradiography following chronic morphine administration to rats, although the molecular mechanism of desensitization was not identified (Sim *et al.*, 1996).

There is now also good evidence to indicate that endogenous levels of PKC play a major role in morphine tolerance *in vivo*. Tolerance to the antinociceptive effects of morphine can be reduced by co-administration of PKC inhibitors (Granados-Soto *et al.*, 2000; Inoue & Ueda, 2000; Bohn *et al.*, 2002; Hua *et al.*, 2002; Smith *et al.*, 2003) or by blockade of various Gq-coupled receptors (Watkins *et al.*, 1984; Dourish *et al.*, 1990; Mitchell *et al.*, 2000; Popik *et al.*, 2000; Powell *et al.*, 2000, 2003; Tortorici *et al.*, 2003). What our *in vitro* and *ex vivo* experiments on brain slices demonstrate is that the site of action of PKC is within the MOPr-expressing neurons themselves, and that PKC activation enhances morphine-induced MOPr desensitization.

In conclusion, we show that morphine can induce robust desensitization of endogenously expressed MOPrs in mature neurons both *in vitro* and *in vivo*, and that this desensitization requires ongoing activation of PKC. Our data support the view that MOPr desensitization by a PKC-dependent mechanism underlies the maintenance of morphine tolerance.

## Abbreviations

aCSF: artificial cerebrospinal fluid
LC: locus coeruleus
MOPr: μ-opioid receptor
NA: noradrenaline
oxo-M: oxotremorine-M
PKC: protein kinase C
PMA: phorbol 12-myristate 13-acetate
β-FNA: β-funaltrexamine

## Supporting Information

Fig. S1. NA and DAMGO responses.
