# Supplementary material for: Role of protein kinase C and μ-opioid receptor (MOPr) desensitization in tolerance to morphine in rat locus coeruleus neurons
Source: Eur J Neurosci. 2009 Jan;29(2):307–18. doi: 10.1111/j.1460-9568.2008.06573.x (PMC2695152; doi:10.1111/j.1460-9568.2008.06573.x)
Supplement: Supplementary file 1 [file ejn0029-0307-SD1.doc]

**
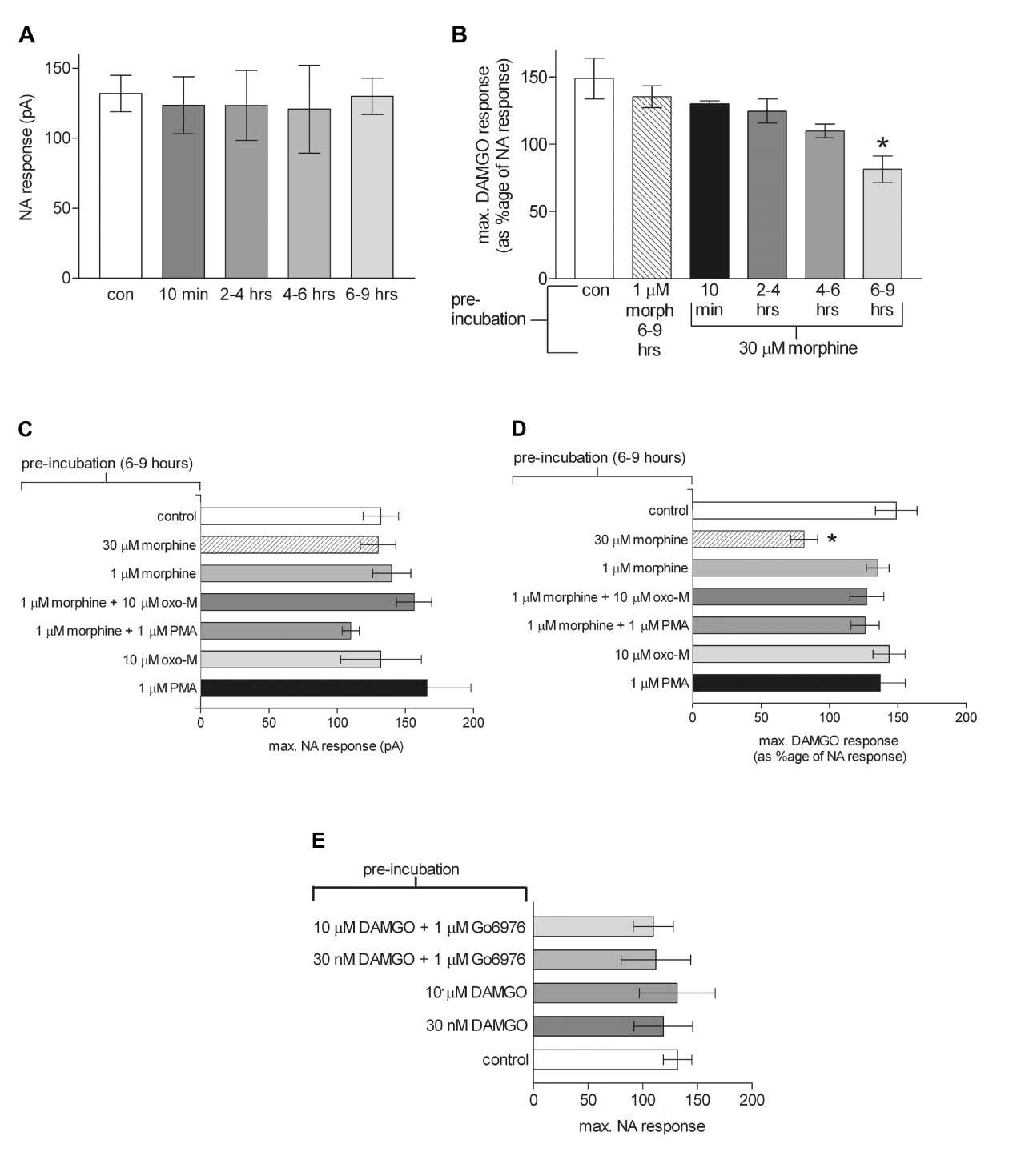
**

**Fig. S1. NA and DAMGO responses.** (A) Amplitudes of noradrenaline (NA: 100 μM) responses following pre-treatment with 30 μM morphine for 10 min, 2-4 h, 4-6 h and 6-9 h are unchanged. (B) Maximum DAMGO responses (10 μM). Experiments are as those shown in Fig. 1E. Maximum DAMGO responses were significantly decreased only after 6-9 h pre-treatment with 30 μM morphine (**P* < 0.05, Student’s *t*-test). (C) Maximum noradrenaline responses (NA: 100 μM) following pre-treatments as shown in Fig. 2B were not different from control. (D) Maximum DAMGO responses (10 μM) following pre-treatments as shown in Fig. 2B. **P* < 0.05, Student’s *t*-test. (E) Maximum noradrenaline (NA: 100 μM) responses following pre-treatments as in Fig. 4E were unchanged from controls. All error bars represent S.E.M.
